# {[Eth­yl(pyridin-4-ylmeth­yl)carbamo­thio­yl]sulfanido-κ^2^ *S*,*S*′}(1,4,7,10,13,16-hexa­oxa­cyclo­octa­decane-κ^6^ *O*)potassium

**DOI:** 10.1107/S1600536813021569

**Published:** 2013-08-07

**Authors:** Hadi D. Arman, Pavel Poplaukhin, Edward R. T. Tiekink

**Affiliations:** a Department of Chemistry, The University of Texas at San Antonio, One UTSA Circle, San Antonio, Texas 78249-0698, USA; b Chemical Abstracts Service, 2540 Olentangy River Rd, Columbus, Ohio 43202, USA; c Department of Chemistry, University of Malaya, 50603 Kuala Lumpur, Malaysia

## Abstract

The asymmetric unit of title salt co-crystal, [K(C_9_H_11_N_2_S_2_)(C_12_H_24_O_6_)], comprises a K^+^ cation, an ^−^S_2_CN(Et)py anion and a 18-crown-6 mol­ecule. Substantial delocalization of π-electron density is evident in the di­thio­carbamate anion, as indicated by the equivalent C—S bond lengths. The K^+^ cation sits within an O_6_S_2_ donor set lying 0.7506 (6) Å out of the least-squares plane through the six O atoms (r.m.s. deviation = 0.1766 Å) of the 18-crown-6 mol­ecule with the two S atoms being on one side of this plane. Supra­molecular layers in the *bc* plane, sustained by C—H⋯O and C—H⋯π inter­actions, feature in the crystal packing.

## Related literature

For the relevance of functionalized di­thio­carbamate ligands, see: Tan *et al.* (2013 ▶). For Cu, Hg and Sn structures of ^−^S_2_CN(Et)py, see: Barba *et al.* (2012 ▶); Singh *et al.* (2011 ▶); Rajput *et al.* (2012 ▶). For a structure featuring a similar coordination geometry for K^+^, see: Harrington *et al.* (2004 ▶).
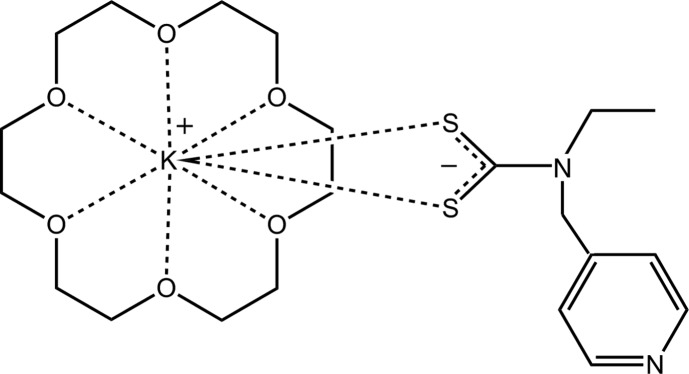


## Experimental

### 

#### Crystal data

[K(C_9_H_11_N_2_S_2_)(C_12_H_24_O_6_)]
*M* *_r_* = 514.73
Monoclinic, 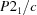

*a* = 17.077 (3) Å
*b* = 17.816 (3) Å
*c* = 8.5107 (17) Å
β = 96.010 (3)°
*V* = 2575.1 (8) Å^3^
*Z* = 4
Mo *K*α radiation
μ = 0.41 mm^−1^
*T* = 98 K
0.50 × 0.40 × 0.08 mm

#### Data collection

Rigaku AFC12/SATURN724 diffractometer
Absorption correction: multi-scan (*ABSCOR*; Higashi, 1995 ▶) *T*_min_ = 0.634, *T*_max_ = 1.000
13965 measured reflections
5807 independent reflections
5310 reflections with *I* > 2σ(*I*)
*R*_int_ = 0.034

#### Refinement

*R*[*F* ^2^ > 2σ(*F* ^2^)] = 0.040
*wR*(*F* ^2^) = 0.100
*S* = 1.09
5807 reflections
290 parameters
H-atom parameters constrained
Δρ_max_ = 0.54 e Å^−3^
Δρ_min_ = −0.26 e Å^−3^

### 

Data collection: *CrystalClear* (Molecular Structure Corporation & Rigaku, 2005 ▶); cell refinement: *CrystalClear*; data reduction: *CrystalClear*; program(s) used to solve structure: *SHELXS97* (Sheldrick, 2008 ▶); program(s) used to refine structure: *SHELXL97* (Sheldrick, 2008 ▶); molecular graphics: *ORTEPII* (Johnson, 1976 ▶) and *DIAMOND* (Brandenburg, 2006 ▶); software used to prepare material for publication: *publCIF* (Westrip, 2010 ▶).

## Supplementary Material

Crystal structure: contains datablock(s) general, I. DOI: 10.1107/S1600536813021569/lh5639sup1.cif

Structure factors: contains datablock(s) I. DOI: 10.1107/S1600536813021569/lh5639Isup2.hkl

Supplementary material file. DOI: 10.1107/S1600536813021569/lh5639Isup3.cml

Additional supplementary materials:  crystallographic information; 3D view; checkCIF report

## Figures and Tables

**Table 1:** Selected bond lengths (Å)

K1—O1	2.7710 (12)
K1—O2	2.9414 (13)
K1—O3	2.8203 (13)
K1—O4	2.9712 (13)
K1—O5	2.8098 (13)
K1—O6	2.9788 (13)
K1—S1	3.1804 (7)
K1—S2	3.2393 (6)
S1—C1	1.7174 (17)
S2—C1	1.7103 (16)
N1—C1	1.362 (2)

**Table 2:** Hydrogen-bond geometry (Å, °) *Cg*1 is the centroid of the N3,C3–C7 ring.

*D*—H⋯*A*	*D*—H	H⋯*A*	*D*⋯*A*	*D*—H⋯*A*
C6—H6⋯O1^i^	0.95	2.51	3.311 (2)	143
C2—H2*B*⋯*Cg*1^ii^	0.99	2.87	3.4275 (18)	116
C12—H12*B*⋯*Cg*1^iii^	0.99	2.95	3.820 (2)	148

Symmetry codes: (i) 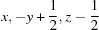; (ii) 
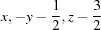
; (iii) 
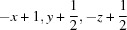
.

**Fig. 1.**
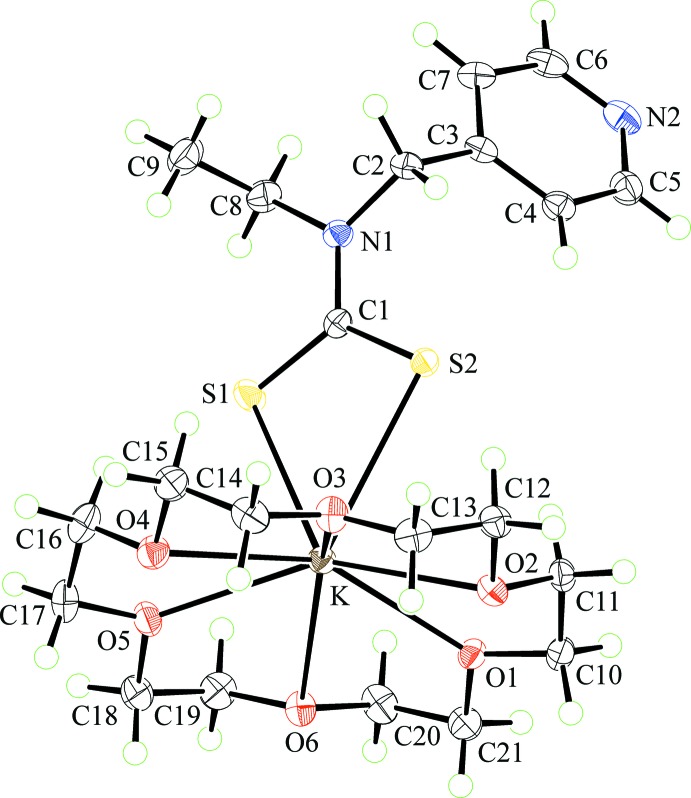
Molecular structures of the components of (I), showing atom-labelling scheme and displacement ellipsoids at the 50% probability level.

**Fig. 2.**
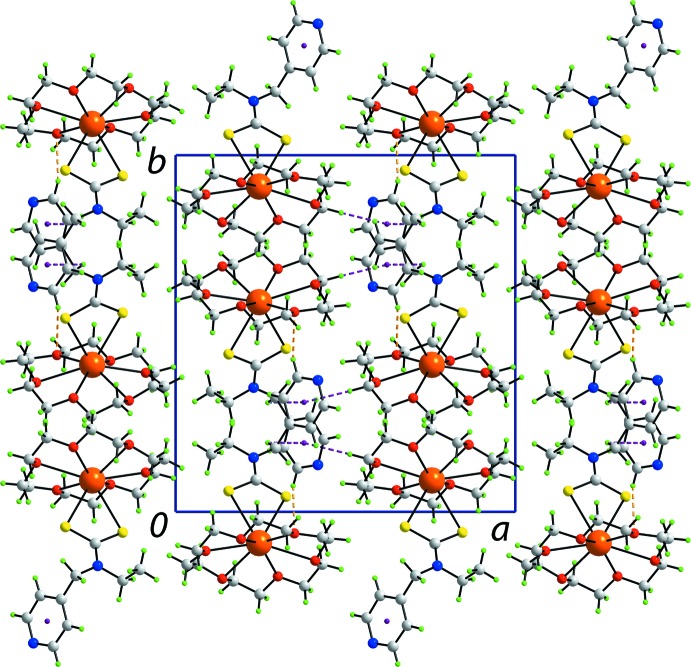
Unit-cell contents in (I) viewed in projection down the c axis. The C—H···O and C—H···π interactions are shown as orange and purple dashed lines, respectively.

## supplementary crystallographic information

### 1. Comment

The supramolecular chemistry of dithiocarbamate (^-^S_2_CN*R*_2_) compounds compared with their xanthate (^-^S_2_CO*R*_2_) counterparts is rather limited owing to the strong chelating ability of the anion, which often precludes the possibility of forming intermolecular M···S interactions (Tan *et al.*, 2013). One way of overcoming this is to functionalize the dithiocarbamate ligand, as in the title salt co-crystal, (I), where the dithiocarbamate ligand carries a pyridyl residue. Crystal structures containing this dithiocarbamate ligand have been reported in recent years (Barba *et al.*, 2012; Singh *et al.*, 2011; Rajput *et al.*, 2012).

The asymmetric unit of (I), Fig. 1, comprises a K^+^ cation, an ^-^S_2_CN(Et)py anion and a 18-crown-6 molecule. The dithiocarbamate ligand exhibits the expected features with equivalent C—S bond lengths and a short C—N bond, Table 1, consistent with a significant contribution of the ^(2-)^S_2_C=N^(+)^(Et)py canonical form to the overall electronic structure. The ethyl and pyridyl substituents lie to either side of the S_2_CN plane, as is normally the case for dithiocarbamate anions.

The K^+^ cation is coordinated by the six oxygen atoms of the 18-crown-6 molecule and the two sulfur atoms of the dithiocarbamate anion, Table 1. The cation lies 0.7506 (6) Å out of the best plane through the six oxygen atoms (r.m.s. deviation = 0.1766 Å) in the direction of the sulfur atoms. The resulting O_6_S_2_ donor set has a precedent in the literature, namely in the structure of [K18-crown-6][Cd(SCN)_3_] (Harrington *et al.*, 2004).

In the crystal packing, molecules assemble into supramolecular layers in the *bc* plane by a combination of pyridyl-C—H···O and methylene-C—H···π interactions, Table 2 and Fig. 2.

### 2. Experimental

K^±^S_2_CN(Et)(CH_2_py) (250 mg,1.00 mmol; prepared from 4-(ethylaminomethyl)pyridine, carbon disulfide and potassium hydroxide *via* the standard route) and 18-crown-6 (264 mg, 1.00 mmol) were dissolved in methanol (25 ml). The solution was filtered and left to evaporate slowly. As the solvent evaporated, the solution turned into yellow oil, from which crystals eventually appeared after about 10 days. Yield – quantitative. IR (cm^-1^): 2888(*s*), 1475(*m*), 1455(*m*), 1350(ms), 1069(*s*), 960(*s*), 836(*s*). NMR ^1^H: δ (p.p.m.) 8.43 (*dd*, Ar, 2.44 Hz, 1.80 Hz), 7.22 (*d*, Ar, 6.30 Hz), 5.46 (*s*, –CH_2_-py), 3.98 (*q*, Et—CH_2_, 6.88 Hz), 3.55 (*s*, 18-crown-6), 1.07 (*t*, Me, 6.89 Hz). *M*.pt: = 409–411 K (uncorrected).

### 3. Refinement

C-bound H-atoms were placed in calculated positions (C—H = 0.95–0.99 Å) and were included in the refinement in the riding model approximation with *U*_iso_(H) set to 1.2–1.5*U*_eq_(C). One reflection, *i.e*. (2 0 0), was omitted from the final refinement owing to poor agreement.

### Crystal data

**Table:**

[K(C_9_H_11_N_2_S_2_)(C_12_H_24_O_6_)]	*F*(000) = 1096
*M**_r_* = 514.73	*D*_x_ = 1.328 Mg m^−^^3^
Monoclinic, *P*2_1_/*c*	Mo *K*α radiation, λ = 0.71069 Å
Hall symbol: -P 2ybc	Cell parameters from 16821 reflections
*a* = 17.077 (3) Å	θ = 2.3–40.7°
*b* = 17.816 (3) Å	µ = 0.41 mm^−^^1^
*c* = 8.5107 (17) Å	*T* = 98 K
β = 96.010 (3)°	Prism, colourless
*V* = 2575.1 (8) Å^3^	0.50 × 0.40 × 0.08 mm
*Z* = 4	

### Data collection

**Table:**

Rigaku AFC12K/SATURN724 diffractometer	5807 independent reflections
Radiation source: fine-focus sealed tube	5310 reflections with *I* > 2σ(*I*)
Graphite monochromator	*R*_int_ = 0.034
ω scans	θ_max_ = 27.5°, θ_min_ = 2.3°
Absorption correction: multi-scan (*ABSCOR*; Higashi, 1995)	*h* = −18→22
*T*_min_ = 0.634, *T*_max_ = 1.000	*k* = −14→23
13965 measured reflections	*l* = −9→11

### Refinement

**Table:**

Refinement on *F*^2^	Primary atom site location: structure-invariant direct methods
Least-squares matrix: full	Secondary atom site location: difference Fourier map
*R*[*F*^2^ > 2σ(*F*^2^)] = 0.040	Hydrogen site location: inferred from neighbouring sites
*wR*(*F*^2^) = 0.100	H-atom parameters constrained
*S* = 1.09	*w* = 1/[σ^2^(*F*_o_^2^) + (0.0443*P*)^2^ + 0.8477*P*] where *P* = (*F*_o_^2^ + 2*F*_c_^2^)/3
5807 reflections	(Δ/σ)_max_ = 0.001
290 parameters	Δρ_max_ = 0.54 e Å^−^^3^
0 restraints	Δρ_min_ = −0.26 e Å^−^^3^

### Special details

**Table:**

Geometry. All s.u.'s (except the s.u. in the dihedral angle between two l.s. planes) are estimated using the full covariance matrix. The cell s.u.'s are taken into account individually in the estimation of s.u.'s in distances, angles and torsion angles; correlations between s.u.'s in cell parameters are only used when they are defined by crystal symmetry. An approximate (isotropic) treatment of cell s.u.'s is used for estimating s.u.'s involving l.s. planes.
Refinement. Refinement of *F*^2^ against ALL reflections. The weighted *R*-factor *wR* and goodness of fit *S* are based on *F*^2^, conventional *R*-factors *R* are based on *F*, with *F* set to zero for negative *F*^2^. The threshold expression of *F*^2^ > 2σ(*F*^2^) is used only for calculating *R*-factors(gt) *etc*. and is not relevant to the choice of reflections for refinement. *R*-factors based on *F*^2^ are statistically about twice as large as those based on *F*, and *R*- factors based on ALL data will be even larger.

### Fractional atomic coordinates and isotropic or equivalent isotropic displacement parameters (Å^2^)

**Table:**

	*x*	*y*	*z*	*U*_iso_*/*U*_eq_	
K1	0.24561 (2)	0.587841 (19)	0.53186 (4)	0.01748 (9)	
S1	0.15616 (3)	0.44193 (3)	0.39265 (6)	0.02666 (11)	
S2	0.32453 (2)	0.45749 (2)	0.33489 (4)	0.01782 (10)	
O1	0.34937 (7)	0.56053 (7)	0.79834 (13)	0.0194 (2)	
O2	0.41075 (7)	0.63733 (7)	0.55515 (13)	0.0207 (2)	
O3	0.30075 (7)	0.68129 (7)	0.29964 (14)	0.0233 (3)	
O4	0.13744 (7)	0.68803 (7)	0.33222 (14)	0.0243 (3)	
O5	0.08784 (7)	0.61018 (7)	0.58646 (14)	0.0242 (3)	
O6	0.19263 (7)	0.57671 (7)	0.85357 (13)	0.0220 (2)	
N1	0.22952 (8)	0.34829 (8)	0.21384 (16)	0.0188 (3)	
N2	0.41879 (10)	0.13141 (9)	0.35162 (18)	0.0261 (3)	
C1	0.23661 (9)	0.41094 (9)	0.30594 (18)	0.0172 (3)	
C2	0.29360 (10)	0.32110 (9)	0.12539 (18)	0.0198 (3)	
H2A	0.3316	0.3624	0.1153	0.024*	
H2B	0.2714	0.3063	0.0177	0.024*	
C3	0.33654 (10)	0.25475 (9)	0.20519 (18)	0.0193 (3)	
C4	0.39809 (10)	0.26495 (10)	0.32451 (19)	0.0215 (3)	
H4	0.4133	0.3141	0.3588	0.026*	
C5	0.43679 (10)	0.20272 (10)	0.3926 (2)	0.0250 (4)	
H5	0.4787	0.2110	0.4733	0.030*	
C6	0.35984 (11)	0.12247 (10)	0.2361 (2)	0.0259 (4)	
H6	0.3460	0.0728	0.2038	0.031*	
C7	0.31778 (11)	0.18124 (10)	0.1607 (2)	0.0240 (3)	
H7	0.2766	0.1713	0.0795	0.029*	
C8	0.15514 (10)	0.30749 (10)	0.1812 (2)	0.0243 (4)	
H8A	0.1249	0.3113	0.2740	0.029*	
H8B	0.1664	0.2538	0.1643	0.029*	
C9	0.10550 (12)	0.33806 (12)	0.0364 (2)	0.0329 (4)	
H9A	0.0566	0.3090	0.0179	0.049*	
H9B	0.1350	0.3340	−0.0560	0.049*	
H9C	0.0929	0.3909	0.0539	0.049*	
C10	0.43213 (10)	0.57113 (10)	0.7992 (2)	0.0222 (3)	
H10A	0.4609	0.5290	0.8551	0.027*	
H10B	0.4484	0.6184	0.8545	0.027*	
C11	0.45080 (10)	0.57442 (9)	0.6314 (2)	0.0213 (3)	
H11A	0.5083	0.5798	0.6283	0.026*	
H11B	0.4336	0.5275	0.5758	0.026*	
C12	0.43101 (10)	0.64712 (10)	0.3982 (2)	0.0234 (3)	
H12A	0.4228	0.5996	0.3384	0.028*	
H12B	0.4871	0.6614	0.4006	0.028*	
C13	0.37967 (11)	0.70785 (10)	0.3196 (2)	0.0255 (4)	
H13A	0.3832	0.7537	0.3858	0.031*	
H13B	0.3973	0.7205	0.2157	0.031*	
C14	0.24825 (11)	0.73107 (11)	0.2105 (2)	0.0273 (4)	
H14A	0.2681	0.7425	0.1080	0.033*	
H14B	0.2439	0.7787	0.2688	0.033*	
C15	0.16952 (11)	0.69396 (12)	0.1837 (2)	0.0288 (4)	
H15A	0.1339	0.7240	0.1089	0.035*	
H15B	0.1750	0.6434	0.1378	0.035*	
C16	0.06537 (11)	0.64719 (12)	0.3188 (2)	0.0304 (4)	
H16A	0.0746	0.5948	0.2865	0.037*	
H16B	0.0272	0.6705	0.2378	0.037*	
C17	0.03337 (10)	0.64813 (11)	0.4759 (2)	0.0292 (4)	
H17A	0.0263	0.7005	0.5105	0.035*	
H17B	−0.0184	0.6227	0.4683	0.035*	
C18	0.06301 (10)	0.60856 (11)	0.7410 (2)	0.0269 (4)	
H18A	0.0068	0.5942	0.7356	0.032*	
H18B	0.0693	0.6588	0.7904	0.032*	
C19	0.11281 (11)	0.55225 (11)	0.8369 (2)	0.0275 (4)	
H19A	0.0939	0.5468	0.9424	0.033*	
H19B	0.1087	0.5027	0.7838	0.033*	
C20	0.24280 (11)	0.52364 (11)	0.9397 (2)	0.0277 (4)	
H20A	0.2398	0.4747	0.8841	0.033*	
H20B	0.2256	0.5162	1.0461	0.033*	
C21	0.32592 (11)	0.55234 (11)	0.95408 (19)	0.0260 (4)	
H21A	0.3291	0.6013	1.0093	0.031*	
H21B	0.3613	0.5166	1.0158	0.031*	

### Atomic displacement parameters (Å^2^)

**Table:**

	*U*^11^	*U*^22^	*U*^33^	*U*^12^	*U*^13^	*U*^23^
K1	0.01821 (17)	0.01634 (17)	0.01786 (17)	0.00049 (12)	0.00177 (12)	−0.00050 (11)
S1	0.0179 (2)	0.0253 (2)	0.0378 (3)	−0.00251 (16)	0.00775 (17)	−0.01033 (18)
S2	0.01652 (19)	0.01639 (19)	0.02050 (19)	−0.00146 (14)	0.00174 (14)	−0.00132 (13)
O1	0.0181 (6)	0.0223 (6)	0.0176 (5)	−0.0003 (5)	0.0003 (4)	0.0013 (4)
O2	0.0209 (6)	0.0199 (6)	0.0221 (6)	0.0013 (5)	0.0053 (4)	0.0009 (4)
O3	0.0239 (6)	0.0211 (6)	0.0249 (6)	−0.0006 (5)	0.0027 (5)	0.0061 (5)
O4	0.0247 (6)	0.0270 (7)	0.0211 (6)	0.0009 (5)	0.0016 (5)	0.0019 (5)
O5	0.0196 (6)	0.0303 (7)	0.0229 (6)	0.0061 (5)	0.0036 (4)	0.0017 (5)
O6	0.0215 (6)	0.0226 (6)	0.0222 (6)	0.0006 (5)	0.0030 (4)	0.0052 (4)
N1	0.0196 (7)	0.0170 (7)	0.0198 (6)	−0.0012 (5)	0.0017 (5)	−0.0016 (5)
N2	0.0321 (8)	0.0197 (7)	0.0282 (8)	0.0049 (6)	0.0105 (6)	0.0018 (6)
C1	0.0183 (7)	0.0159 (7)	0.0171 (7)	−0.0007 (6)	−0.0001 (5)	0.0023 (5)
C2	0.0246 (8)	0.0183 (8)	0.0170 (7)	0.0009 (6)	0.0041 (6)	−0.0008 (6)
C3	0.0232 (8)	0.0170 (8)	0.0188 (7)	0.0012 (6)	0.0073 (6)	−0.0012 (6)
C4	0.0234 (8)	0.0176 (8)	0.0241 (8)	−0.0013 (6)	0.0054 (6)	−0.0014 (6)
C5	0.0247 (9)	0.0254 (9)	0.0249 (8)	0.0039 (7)	0.0033 (6)	0.0013 (7)
C6	0.0374 (10)	0.0147 (8)	0.0273 (9)	−0.0002 (7)	0.0113 (7)	−0.0023 (6)
C7	0.0321 (9)	0.0199 (8)	0.0204 (8)	−0.0009 (7)	0.0051 (6)	−0.0038 (6)
C8	0.0252 (9)	0.0182 (8)	0.0293 (9)	−0.0057 (7)	0.0011 (7)	−0.0029 (6)
C9	0.0279 (10)	0.0348 (10)	0.0341 (10)	−0.0052 (8)	−0.0062 (7)	−0.0040 (8)
C10	0.0194 (8)	0.0196 (8)	0.0264 (8)	−0.0001 (6)	−0.0039 (6)	0.0002 (6)
C11	0.0151 (7)	0.0185 (8)	0.0300 (8)	0.0004 (6)	0.0011 (6)	−0.0014 (6)
C12	0.0210 (8)	0.0270 (9)	0.0232 (8)	−0.0029 (7)	0.0077 (6)	−0.0008 (7)
C13	0.0283 (9)	0.0247 (9)	0.0244 (8)	−0.0068 (7)	0.0072 (7)	0.0027 (7)
C14	0.0361 (10)	0.0241 (9)	0.0224 (8)	0.0067 (8)	0.0070 (7)	0.0075 (7)
C15	0.0316 (9)	0.0367 (10)	0.0178 (8)	0.0080 (8)	0.0004 (7)	0.0033 (7)
C16	0.0266 (9)	0.0347 (10)	0.0279 (9)	−0.0007 (8)	−0.0074 (7)	0.0045 (7)
C17	0.0167 (8)	0.0321 (10)	0.0378 (10)	0.0019 (7)	−0.0007 (7)	0.0050 (8)
C18	0.0199 (8)	0.0340 (10)	0.0279 (9)	−0.0010 (7)	0.0079 (6)	−0.0011 (7)
C19	0.0255 (9)	0.0325 (10)	0.0257 (9)	−0.0066 (8)	0.0080 (7)	0.0035 (7)
C20	0.0320 (10)	0.0272 (9)	0.0250 (8)	0.0062 (8)	0.0080 (7)	0.0110 (7)
C21	0.0298 (9)	0.0309 (9)	0.0172 (8)	0.0068 (8)	0.0016 (6)	0.0041 (6)

### Geometric parameters (Å, º)

**Table:**

K1—O1	2.7710 (12)	C7—H7	0.9500
K1—O2	2.9414 (13)	C8—C9	1.522 (3)
K1—O3	2.8203 (13)	C8—H8A	0.9900
K1—O4	2.9712 (13)	C8—H8B	0.9900
K1—O5	2.8098 (13)	C9—H9A	0.9800
K1—O6	2.9788 (13)	C9—H9B	0.9800
K1—S1	3.1804 (7)	C9—H9C	0.9800
K1—S2	3.2393 (6)	C10—C11	1.497 (2)
S1—C1	1.7174 (17)	C10—H10A	0.9900
S2—C1	1.7103 (16)	C10—H10B	0.9900
O1—C10	1.425 (2)	C11—H11A	0.9900
O1—C21	1.432 (2)	C11—H11B	0.9900
O2—C12	1.425 (2)	C12—C13	1.505 (3)
O2—C11	1.433 (2)	C12—H12A	0.9900
O3—C13	1.422 (2)	C12—H12B	0.9900
O3—C14	1.422 (2)	C13—H13A	0.9900
O4—C16	1.424 (2)	C13—H13B	0.9900
O4—C15	1.433 (2)	C14—C15	1.494 (3)
O5—C17	1.423 (2)	C14—H14A	0.9900
O5—C18	1.424 (2)	C14—H14B	0.9900
O6—C19	1.424 (2)	C15—H15A	0.9900
O6—C20	1.426 (2)	C15—H15B	0.9900
N1—C1	1.362 (2)	C16—C17	1.497 (3)
N1—C8	1.465 (2)	C16—H16A	0.9900
N1—C2	1.474 (2)	C16—H16B	0.9900
N2—C6	1.341 (2)	C17—H17A	0.9900
N2—C5	1.344 (2)	C17—H17B	0.9900
C2—C3	1.514 (2)	C18—C19	1.500 (3)
C2—H2A	0.9900	C18—H18A	0.9900
C2—H2B	0.9900	C18—H18B	0.9900
C3—C7	1.391 (2)	C19—H19A	0.9900
C3—C4	1.395 (2)	C19—H19B	0.9900
C4—C5	1.386 (2)	C20—C21	1.502 (3)
C4—H4	0.9500	C20—H20A	0.9900
C5—H5	0.9500	C20—H20B	0.9900
C6—C7	1.388 (3)	C21—H21A	0.9900
C6—H6	0.9500	C21—H21B	0.9900
			
O1—K1—O5	115.56 (4)	C9—C8—H8A	109.2
O1—K1—O3	116.45 (4)	N1—C8—H8B	109.2
O5—K1—O3	115.37 (4)	C9—C8—H8B	109.2
O1—K1—O2	58.07 (3)	H8A—C8—H8B	107.9
O5—K1—O2	151.10 (4)	C8—C9—H9A	109.5
O3—K1—O2	58.82 (3)	C8—C9—H9B	109.5
O1—K1—O4	151.58 (4)	H9A—C9—H9B	109.5
O5—K1—O4	57.31 (4)	C8—C9—H9C	109.5
O3—K1—O4	58.52 (4)	H9A—C9—H9C	109.5
O2—K1—O4	113.13 (4)	H9B—C9—H9C	109.5
O1—K1—O6	57.71 (4)	O1—C10—C11	108.09 (12)
O5—K1—O6	57.90 (3)	O1—C10—H10A	110.1
O3—K1—O6	147.06 (4)	C11—C10—H10A	110.1
O2—K1—O6	109.84 (3)	O1—C10—H10B	110.1
O4—K1—O6	109.58 (4)	C11—C10—H10B	110.1
O1—K1—S1	113.72 (3)	H10A—C10—H10B	108.4
O5—K1—S1	75.28 (3)	O2—C11—C10	108.88 (13)
O3—K1—S1	113.83 (3)	O2—C11—K1	54.74 (7)
O2—K1—S1	133.61 (3)	C10—C11—K1	85.77 (9)
O4—K1—S1	91.86 (3)	O2—C11—H11A	109.9
O6—K1—S1	96.19 (3)	C10—C11—H11A	109.9
O1—K1—S2	91.87 (3)	K1—C11—H11A	162.0
O5—K1—S2	130.18 (3)	O2—C11—H11B	109.9
O3—K1—S2	82.71 (3)	C10—C11—H11B	109.9
O2—K1—S2	78.57 (3)	K1—C11—H11B	72.8
O4—K1—S2	113.72 (3)	H11A—C11—H11B	108.3
O6—K1—S2	127.65 (3)	O2—C12—C13	108.48 (14)
S1—K1—S2	55.310 (15)	O2—C12—H12A	110.0
O1—K1—C11	42.03 (4)	C13—C12—H12A	110.0
O5—K1—C11	156.33 (4)	O2—C12—H12B	110.0
O3—K1—C11	79.17 (4)	C13—C12—H12B	110.0
O2—K1—C11	23.43 (4)	H12A—C12—H12B	108.4
O4—K1—C11	136.13 (4)	O3—C13—C12	108.36 (14)
O6—K1—C11	99.47 (4)	O3—C13—H13A	110.0
S1—K1—C11	117.42 (3)	C12—C13—H13A	110.0
S2—K1—C11	67.60 (3)	O3—C13—H13B	110.0
C1—S1—K1	92.77 (6)	C12—C13—H13B	110.0
C1—S2—K1	90.90 (6)	H13A—C13—H13B	108.4
C10—O1—C21	112.46 (12)	O3—C14—C15	108.20 (15)
C10—O1—K1	121.99 (9)	O3—C14—H14A	110.1
C21—O1—K1	123.97 (10)	C15—C14—H14A	110.1
C12—O2—C11	111.66 (13)	O3—C14—H14B	110.1
C12—O2—K1	107.38 (9)	C15—C14—H14B	110.1
C11—O2—K1	101.83 (9)	H14A—C14—H14B	108.4
C13—O3—C14	113.14 (13)	O4—C15—C14	108.61 (14)
C13—O3—K1	119.63 (9)	O4—C15—H15A	110.0
C14—O3—K1	120.25 (10)	C14—C15—H15A	110.0
C16—O4—C15	111.96 (14)	O4—C15—H15B	110.0
C16—O4—K1	102.67 (10)	C14—C15—H15B	110.0
C15—O4—K1	106.05 (10)	H15A—C15—H15B	108.3
C17—O5—C18	112.52 (14)	O4—C16—C17	108.42 (15)
C17—O5—K1	122.00 (10)	O4—C16—H16A	110.0
C18—O5—K1	122.23 (10)	C17—C16—H16A	110.0
C19—O6—C20	111.44 (14)	O4—C16—H16B	110.0
C19—O6—K1	107.97 (9)	C17—C16—H16B	110.0
C20—O6—K1	107.01 (9)	H16A—C16—H16B	108.4
C1—N1—C8	122.62 (14)	O5—C17—C16	108.10 (15)
C1—N1—C2	122.51 (14)	O5—C17—H17A	110.1
C8—N1—C2	114.55 (13)	C16—C17—H17A	110.1
C6—N2—C5	115.85 (15)	O5—C17—H17B	110.1
N1—C1—S2	120.28 (12)	C16—C17—H17B	110.1
N1—C1—S1	118.94 (12)	H17A—C17—H17B	108.4
S2—C1—S1	120.78 (9)	O5—C18—C19	108.02 (14)
N1—C2—C3	112.22 (13)	O5—C18—H18A	110.1
N1—C2—H2A	109.2	C19—C18—H18A	110.1
C3—C2—H2A	109.2	O5—C18—H18B	110.1
N1—C2—H2B	109.2	C19—C18—H18B	110.1
C3—C2—H2B	109.2	H18A—C18—H18B	108.4
H2A—C2—H2B	107.9	O6—C19—C18	109.37 (14)
C7—C3—C4	117.07 (16)	O6—C19—H19A	109.8
C7—C3—C2	121.74 (15)	C18—C19—H19A	109.8
C4—C3—C2	121.18 (15)	O6—C19—H19B	109.8
C5—C4—C3	119.35 (16)	C18—C19—H19B	109.8
C5—C4—H4	120.3	H19A—C19—H19B	108.2
C3—C4—H4	120.3	O6—C20—C21	109.15 (14)
N2—C5—C4	124.15 (16)	O6—C20—H20A	109.9
N2—C5—H5	117.9	C21—C20—H20A	109.9
C4—C5—H5	117.9	O6—C20—H20B	109.9
N2—C6—C7	124.17 (16)	C21—C20—H20B	109.9
N2—C6—H6	117.9	H20A—C20—H20B	108.3
C7—C6—H6	117.9	O1—C21—C20	108.22 (14)
C6—C7—C3	119.41 (16)	O1—C21—H21A	110.1
C6—C7—H7	120.3	C20—C21—H21A	110.1
C3—C7—H7	120.3	O1—C21—H21B	110.1
N1—C8—C9	112.00 (15)	C20—C21—H21B	110.1
N1—C8—H8A	109.2	H21A—C21—H21B	108.4
			
O1—K1—S1—C1	−77.63 (6)	O4—K1—O5—C18	−147.58 (14)
O5—K1—S1—C1	170.54 (6)	O6—K1—O5—C18	3.16 (12)
O3—K1—S1—C1	58.93 (6)	S1—K1—O5—C18	110.39 (13)
O2—K1—S1—C1	−9.91 (7)	S2—K1—O5—C18	117.62 (12)
O4—K1—S1—C1	115.09 (6)	C11—K1—O5—C18	−15.54 (19)
O6—K1—S1—C1	−134.99 (6)	O1—K1—O6—C19	−152.93 (12)
S2—K1—S1—C1	−2.75 (5)	O5—K1—O6—C19	29.60 (10)
C11—K1—S1—C1	−30.95 (6)	O3—K1—O6—C19	117.44 (11)
O1—K1—S2—C1	120.60 (6)	O2—K1—O6—C19	−179.66 (10)
O5—K1—S2—C1	−5.75 (6)	O4—K1—O6—C19	55.48 (11)
O3—K1—S2—C1	−122.97 (6)	S1—K1—O6—C19	−38.71 (11)
O2—K1—S2—C1	177.47 (6)	S2—K1—O6—C19	−88.96 (11)
O4—K1—S2—C1	−72.11 (6)	C11—K1—O6—C19	−157.90 (11)
O6—K1—S2—C1	71.14 (6)	O1—K1—O6—C20	−32.86 (10)
S1—K1—S2—C1	2.76 (5)	O5—K1—O6—C20	149.67 (12)
C11—K1—S2—C1	155.78 (6)	O3—K1—O6—C20	−122.49 (11)
O5—K1—O1—C10	−160.07 (11)	O2—K1—O6—C20	−59.59 (11)
O3—K1—O1—C10	−19.85 (12)	O4—K1—O6—C20	175.56 (10)
O2—K1—O1—C10	−12.35 (11)	S1—K1—O6—C20	81.36 (11)
O4—K1—O1—C10	−92.03 (13)	S2—K1—O6—C20	31.11 (12)
O6—K1—O1—C10	−162.45 (12)	C11—K1—O6—C20	−37.83 (11)
S1—K1—O1—C10	115.52 (11)	C8—N1—C1—S2	−178.15 (12)
S2—K1—O1—C10	62.94 (11)	C2—N1—C1—S2	−5.0 (2)
C11—K1—O1—C10	10.22 (10)	C8—N1—C1—S1	2.1 (2)
O5—K1—O1—C21	4.51 (13)	C2—N1—C1—S1	175.24 (11)
O3—K1—O1—C21	144.74 (12)	K1—S2—C1—N1	175.37 (12)
O2—K1—O1—C21	152.23 (13)	K1—S2—C1—S1	−4.89 (9)
O4—K1—O1—C21	72.56 (15)	K1—S1—C1—N1	−175.27 (12)
O6—K1—O1—C21	2.14 (11)	K1—S1—C1—S2	4.99 (9)
S1—K1—O1—C21	−79.90 (12)	C1—N1—C2—C3	102.48 (17)
S2—K1—O1—C21	−132.48 (12)	C8—N1—C2—C3	−83.88 (17)
C11—K1—O1—C21	174.81 (14)	N1—C2—C3—C7	96.36 (18)
O1—K1—O2—C12	157.70 (11)	N1—C2—C3—C4	−85.12 (19)
O5—K1—O2—C12	−116.71 (11)	C7—C3—C4—C5	−0.3 (2)
O3—K1—O2—C12	−30.15 (10)	C2—C3—C4—C5	−178.85 (15)
O4—K1—O2—C12	−52.90 (10)	C6—N2—C5—C4	0.7 (3)
O6—K1—O2—C12	−175.69 (10)	C3—C4—C5—N2	−0.3 (3)
S1—K1—O2—C12	64.19 (11)	C5—N2—C6—C7	−0.5 (3)
S2—K1—O2—C12	58.19 (10)	N2—C6—C7—C3	0.0 (3)
C11—K1—O2—C12	117.43 (14)	C4—C3—C7—C6	0.4 (2)
O1—K1—O2—C11	40.26 (9)	C2—C3—C7—C6	179.00 (15)
O5—K1—O2—C11	125.86 (10)	C1—N1—C8—C9	88.48 (19)
O3—K1—O2—C11	−147.58 (10)	C2—N1—C8—C9	−85.15 (18)
O4—K1—O2—C11	−170.34 (9)	C21—O1—C10—C11	176.67 (14)
O6—K1—O2—C11	66.88 (9)	K1—O1—C10—C11	−17.13 (17)
S1—K1—O2—C11	−53.24 (10)	C12—O2—C11—C10	175.41 (13)
S2—K1—O2—C11	−59.24 (9)	K1—O2—C11—C10	−70.29 (12)
O1—K1—O3—C13	3.60 (13)	C12—O2—C11—K1	−114.30 (12)
O5—K1—O3—C13	143.90 (11)	O1—C10—C11—O2	61.78 (17)
O2—K1—O3—C13	−3.84 (11)	O1—C10—C11—K1	11.35 (11)
O4—K1—O3—C13	151.51 (13)	O1—K1—C11—O2	−124.99 (11)
O6—K1—O3—C13	74.36 (14)	O5—K1—C11—O2	−102.72 (12)
S1—K1—O3—C13	−131.72 (11)	O3—K1—C11—O2	27.84 (9)
S2—K1—O3—C13	−84.86 (12)	O4—K1—C11—O2	12.87 (11)
C11—K1—O3—C13	−16.37 (12)	O6—K1—C11—O2	−118.71 (9)
O1—K1—O3—C14	−145.18 (11)	S1—K1—C11—O2	139.19 (8)
O5—K1—O3—C14	−4.88 (13)	S2—K1—C11—O2	114.34 (9)
O2—K1—O3—C14	−152.62 (13)	O1—K1—C11—C10	−8.26 (8)
O4—K1—O3—C14	2.73 (11)	O5—K1—C11—C10	14.00 (16)
O6—K1—O3—C14	−74.42 (14)	O3—K1—C11—C10	144.56 (10)
S1—K1—O3—C14	79.50 (12)	O2—K1—C11—C10	116.72 (14)
S2—K1—O3—C14	126.36 (12)	O4—K1—C11—C10	129.59 (9)
C11—K1—O3—C14	−165.15 (12)	O6—K1—C11—C10	−1.99 (10)
O1—K1—O4—C16	−123.50 (11)	S1—K1—C11—C10	−104.08 (9)
O5—K1—O4—C16	−39.72 (10)	S2—K1—C11—C10	−128.93 (10)
O3—K1—O4—C16	148.45 (11)	C11—O2—C12—C13	173.23 (13)
O2—K1—O4—C16	171.28 (10)	K1—O2—C12—C13	62.42 (14)
O6—K1—O4—C16	−65.79 (11)	C14—O3—C13—C12	−173.42 (14)
S1—K1—O4—C16	31.44 (10)	K1—O3—C13—C12	35.72 (17)
S2—K1—O4—C16	84.02 (10)	O2—C12—C13—O3	−67.22 (17)
C11—K1—O4—C16	165.76 (10)	C13—O3—C14—C15	174.41 (14)
O1—K1—O4—C15	118.89 (12)	K1—O3—C14—C15	−34.93 (17)
O5—K1—O4—C15	−157.34 (12)	C16—O4—C15—C14	−174.80 (15)
O3—K1—O4—C15	30.84 (10)	K1—O4—C15—C14	−63.57 (15)
O2—K1—O4—C15	53.67 (11)	O3—C14—C15—O4	67.93 (18)
O6—K1—O4—C15	176.59 (10)	C15—O4—C16—C17	−176.24 (15)
S1—K1—O4—C15	−86.17 (11)	K1—O4—C16—C17	70.42 (15)
S2—K1—O4—C15	−33.60 (11)	C18—O5—C17—C16	179.48 (15)
C11—K1—O4—C15	48.14 (13)	K1—O5—C17—C16	19.4 (2)
O1—K1—O5—C17	158.90 (12)	O4—C16—C17—O5	−63.1 (2)
O3—K1—O5—C17	18.24 (14)	C17—O5—C18—C19	166.24 (15)
O2—K1—O5—C17	89.17 (14)	K1—O5—C18—C19	−33.78 (19)
O4—K1—O5—C17	10.52 (12)	C20—O6—C19—C18	−178.07 (14)
O6—K1—O5—C17	161.27 (14)	K1—O6—C19—C18	−60.82 (15)
S1—K1—O5—C17	−91.50 (13)	O5—C18—C19—O6	64.13 (19)
S2—K1—O5—C17	−84.27 (13)	C19—O6—C20—C21	−179.81 (14)
C11—K1—O5—C17	142.57 (13)	K1—O6—C20—C21	62.36 (15)
O1—K1—O5—C18	0.79 (14)	C10—O1—C21—C20	−166.78 (14)
O3—K1—O5—C18	−139.87 (12)	K1—O1—C21—C20	27.35 (18)
O2—K1—O5—C18	−68.93 (15)	O6—C20—C21—O1	−61.03 (18)

### Hydrogen-bond geometry (Å, º)

Cg1 is the centroid of the N3,C3–C7 ring.

**Table:**

*D*—H···*A*	*D*—H	H···*A*	*D*···*A*	*D*—H···*A*
C6—H6···O1^i^	0.95	2.51	3.311 (2)	143
C2—H2*B*···*Cg*1^ii^	0.99	2.87	3.4275 (18)	116
C12—H12*B*···*Cg*1^iii^	0.99	2.95	3.820 (2)	148

Symmetry codes: (i) *x*, −*y*+1/2, *z*−1/2; (ii) *x*, −*y*−1/2, *z*−3/2; (iii) −*x*+1, *y*+1/2, −*z*+1/2.
